# Experimental investigation of usage of POE lubricants with Al_2_O_3_, graphene or CNT nanoparticles in a refrigeration compressor

**DOI:** 10.3762/bjnano.14.86

**Published:** 2023-11-02

**Authors:** Kayhan Dağıdır, Kemal Bilen

**Affiliations:** 1 Department of Mechanical Engineering, Tarsus University, Mersin, Turkey; 2 Department of Mechanical Engineering, Ankara Yıldırım Beyazıt University, Ankara, Turkey

**Keywords:** Al_2_O_3_, carbon nanotubes, graphene, nanolubricant, polyolester oil, refrigeration compressor

## Abstract

In this study, the use of nanolubricants containing Al_2_O_3_, graphene, and carbon nanotubes (CNTs) at different mass fractions in a refrigeration compressor was experimentally investigated. The required electrical power of the compressor was measured to determine the effect of the use of nanolubricants. Nanoparticles used in the preparation of nanolubricants were gradually added to the lubricant to determine the optimum nanoparticle mass fraction for each nanoparticle type. Thus, it was found that the compressor operated safely and efficiently with nanolubricants. Furthermore, the optimum mass fractions were determined to be 0.750% for Al_2_O_3_, 0.250% for graphene, and 0.250% for CNTs for operating conditions of this study. As a result, the required electrical power of the compressor decreased by 6.26, 6.82, and 5.55% with the addition of Al_2_O_3_, graphene, and CNT nanoparticles at optimum mass fractions of 0.750, 0.250, and 0.250% to the lubricant, respectively, compared to the compressor using pure oil. Moreover, density and dynamic viscosity of the nanolubricant samples used in the experiments were also measured, and their kinematic viscosity, which is an important parameter for lubricants, was calculated. It was determined that the kinematic viscosity continuously increased with increasing nanoparticle fraction. In conclusion, nanolubricants containing nanoparticles above the optimum mass fraction increase the required electrical power of the compressor. It is concluded that nanoparticle fractions should not be used above the optimum value in nanolubricant applications.

## Introduction

Compressor performance is directly related to the thermophysical properties of the lubricant. Improving the thermophysical properties of lubricants can be tried as a method to improve compressor performance. It is stated that the addition of nanoparticles to lubricants improves their thermophysical properties. Lubricants with nanoparticles are specially called nanolubricants. Shrivastava and Chhalotre [1] conducted various experiments on a refrigeration compressor using a nanolubricant with Al_2_O_3_ nanoparticles at different concentrations. They found that the addition of nanoparticles enhanced the thermophysical properties and heat transfer characteristics of the lubricant. The researchers specified that nanolubricants typically provide greater thermal conductivity and viscosity in comparison to pure lubricants [2]. Sanukrishna and Prakash [3] experimentally investigated the thermophysical properties of a nanolubricant containing TiO_2_ nanoparticles for volume fractions of 0.07 to 0.8% in a temperature range of 20 to 90 °C. The results showed that the thermal conductivity and viscosity of the nanolubricant increased with the increase in volume fraction at a constant temperature. Also, Wadi et al. [4] experimentally examined the thermophysical properties of the nanolubricant containing graphene nanoparticles for mass fractions of 0.025 to 0.5% in a temperature range of 25 to 70 °C. The results showed that the thermal conductivity and viscosity of the nanolubricant increased with the increase in mass fraction at a constant temperature.

This capability in thermal conductivity enhancement can aid in addressing heat transfer issues within systems. Due to the fact that heat transfer takes place at the interface between particles and the liquid in a solid–liquid suspension, enhancing the interfacial area can result in more effective thermal transport properties. Smaller particle sizes result in higher surface-to-volume ratios, consequently raising the thermal conductivity of the liquid. This is attributed to the fact that heat transfer is fundamentally a surface-related process [5]. Nanolubricants have been widely used in recent years to improve the performance of refrigeration compressors [6–8]. Singh et al. [9] experimentally verified the effect of addition of multiwalled carbon nanotube (MWCNT) nanoparticles to a lubricant in a refrigeration compressor. Results showed that the compressor energy consumption decreased up to 31% with the addition of MWCNTs at 0.5% in mass to the lubricant compared to that of the base case. Additionally, Subhedar et al. [10] experimentally investigated the refrigeration compressor by using a nanolubricant containing Al_2_O_3_ nanoparticles. Results showed that the required compressor power decreased up to 27% with the addition of Al_2_O_3_ at 0.075% in volume to the lubricant compared to that of the base case. The most preferred nanoparticles for nanolubricant applications in refrigeration compressors are metal oxides and carbon-based nanoparticles [11–12]. It is emphasized that both kinds of nanoparticles have positive effects on system performance. Krishnan et al. [13]. examined the effects of addition of metal oxide nanoparticles of Al_2_O_3_, SiO_2_, ZrO_2_, and carbon-based nanoparticles of CNTs to the refrigeration compressor lubricant. The study reported that the compressor performance was increased with the addition of both metal oxide and carbon-based nanoparticles to the lubricant compared to that of the base case. In particular, the use of carbon-based nanoparticles is becoming more and more common due to their unique thermal properties [14]. Akkaya et al. [15] experimentally explored the lubrication properties of sepiolite (SP) and its carbon composites carbon black (CB), MWCNT, and reduced graphene oxide (rGO) in a refrigeration compressor. Consequently, the addition of SP-rGO nanoparticles showed the best improvement in the compressor performance. According to the studies in the literature, the usage of nanolubricants in refrigeration compressors reduces energy consumption and provides a positive effect on compressor performance. Some studies in the literature reporting improvement in compressor performance are given in Table 1. Accordingly, it can be noted that the compressor energy consumption reduces mediately 30% with the use of nanolubricants.

**Table 1:** Some studies reporting improvement in compressor performance upon use of nanolubricants.

Reference	Nanoparticle	Findings

Singh et al. [9]	MWCNT	compressor energy consumption decreased up to 31%
Subhedar et al. [10]	Al_2_O_3_	required compressor power decreased up to 27%
Kumar and Elansezhian [16]	ZnO	energy consumption decreased by 21%
Senthilkumar and Praveen [17]	CuO	energy consumption reduced about 11.83%
Harichandran et al. [18]	h-BN	energy consumption decreased up to 60%
Adelekan et al. [19]	TiO_2_	compressor work input decreased up to 50%
Akkaya et al. [20]	Al_2_O_3_	required compressor power decreased up to 12.53%

The literature shows that metal oxides and carbon-based nanoparticles are mainly preferred in nanolubricants used in refrigeration compressors [12]. Therefore, three different types of nanoparticles were used to increase the diversity within the scope of this study. These nanoparticles are metal-oxide-based Al_2_O_3_ and carbon-based (graphene and CNTs) nanoparticles. These nanoparticles are the most preferred nanoparticles in the literature, and they have generally provided the highest enhancement in nanolubricant performance [5]. However, they have not yet been used as nanoparticles in nanolubricant applications in refrigeration compressor using the R1234yf refrigerant. Also, there is no study that shows the determination of optimum fractions of these nanoparticles in nanolubricant applications, although they are widely used in the literature. In addition, in this study, optimum mass fractions were also determined for these types of nanoparticles in nanolubricants used in refrigeration compressors. The determination of the optimum mass fractions was based on the lowest mass fraction determined from similar studies in the literature. The nanoparticles were gradually added to the lubricant starting at the lowest fraction, and the optimum mass fraction of each nanoparticle was determined. A two-step method was used to add nanoparticles to the lubricant. Elimination of precipitation and agglomeration problems of nanoparticles is crucial for nanolubricant applications. On this critical issue in the nanolubricant preparation process, the authors benefited from their previous study [21], which included the basic principles of stable preparation of a nanolubricant containing Al_2_O_3_ as nanoparticles. It was considered that these optimum mass fractions would contribute to the literature. In addition, the kinematic viscosity of the nanolubricants was calculated to understand the situation limiting the nanoparticle fraction in nanolubricant applications. Also, a correlation based on experimental results for each nanoparticle type was proposed for the kinematic viscosity, one of the most important properties of lubricants. The aim of this correlation was to contribute to the studies of researchers who do not have the opportunity to carry out experimental work on nanorefrigerants.

## Methodology

In this study, the effect of using nanolubricant containing Al_2_O_3_, graphene, and CNT nanoparticles at various mass fractions on the performance of a semi-hermetic reciprocating refrigerant compressor was experimentally investigated. Details of the experimental investigation are presented in the following sections.

### Experimental setup

**Figure 1:**
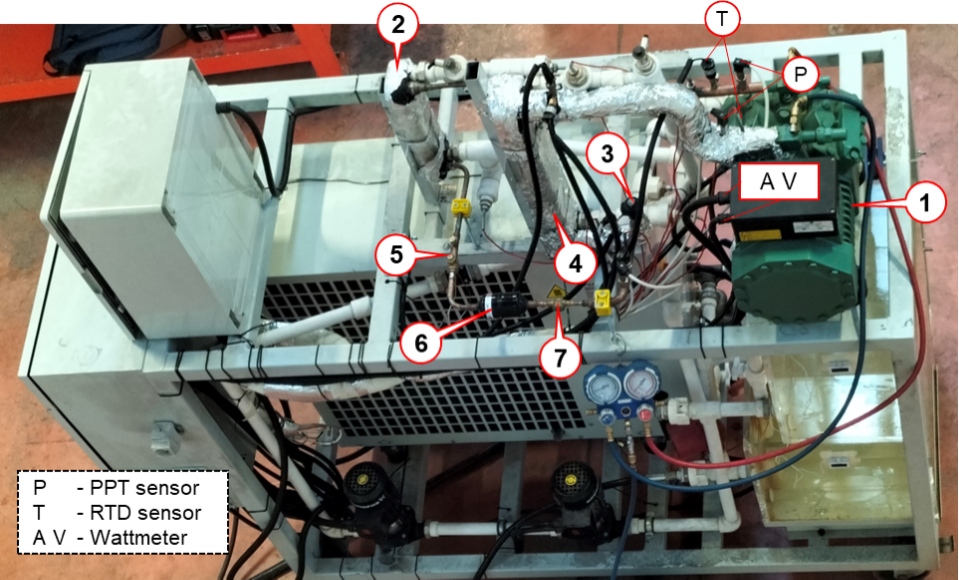
The experimental setup. The components of the system are as follows: 1. Compressor: BITZER 4CES-6Y-40S semi-hermetic reciprocating compressor. 2. Condenser: ALFA LAVAL AC-30EQ-20H-F plate heat exchanger. 3. Expansion valve: DANFOSS ETS 6 electronic valve. 4. Evaporator: ALFA LAVAL AC-70X-20M-F plate heat exchanger. 5. Shut-off valve: DANFOSS GBC manual valve. 6. Filter-drier: DANFOSS DML device. 7. Sight glass: DANFOSS SGP device.

The experimental setup includes a semi-hermetic reciprocating compressor. This compressor is compatible with the refrigerant R1234yf. The experimental setup is shown in Figure 1.

Pressure and temperature measurement devices were placed to control operating conditions at the inlet and outlet of the compressor. Pressure and temperature values were measured at the inlet and outlet to ensure safe operation of the refrigeration compressor. Piezoresistive pressure transmitters (PPT) and resistance temperature detector (RTD) sensors were used for pressure and temperature measurements, respectively. Besides, required electrical power of the compressor was measured using a digital wattmeter. During the tests, the pressure and temperatures at the compressor inlet and outlet were kept approximately constant. The ambient temperature was also controlled during the experiments using a negative temperature coefficient (NTC) thermistor. The technical specifications of the measurement devices used in the experiments are given in Table 2.

**Table 2:** Technical specifications of measurement devices used in the experiments.

Measurement device	Measurement range	Accuracy

PPT pressure gauges	0–25 bar	±0.25%
RTD temperature sensor	‒70–200 °C	±0.5 °C
NTC thermistor	‒20–60 °C	±0.5 °C
Wattmeter	0–10 kW	±1%

### Preparation of the nanolubricant

In this study, nanoparticles of Al_2_O_3_, graphene, and CNTs were added to the refrigeration compressor lubricant (i.e., polyolester, POE, oil) to improve the compressor performance. These nanoparticles were purchased from a manufacturer called Nanografi. Catalog information provided by the manufacturer is given in Table 3. The characterization of the nanoparticles is presented separately in the subsequent sections to verify the catalog information provided by the manufacturer. In the characterization of the nanoparticles used in the study, field-emission scanning electron microscopy (FE-SEM), energy-dispersive X-ray spectroscopy (EDS), and X-ray diffraction (XRD) analyses were performed.

**Table 3:** Catalog information of nanoparticles provided by the Nanografi manufacturer (website: https://nanografi.com/).

Property	Al_2_O_3_	Graphene	CNTs

type	gamma	nanoplatelet	multiwalled
purity	99.5%	99.9%	>96%
color	white	gray	black
average particle size	18 nm	–	–
thickness	–	5 nm	–
diameter	–	18 μm	–
outside diameter	–	–	8–18 nm
inside diameter	–	–	5–10 nm
length	–	–	10–30 μm
specific surface area	140 m^2^/g	170 m^2^/g	220 m^2^/g

### Characterization of Al_2_O_3_ nanoparticles

**Figure 2:**
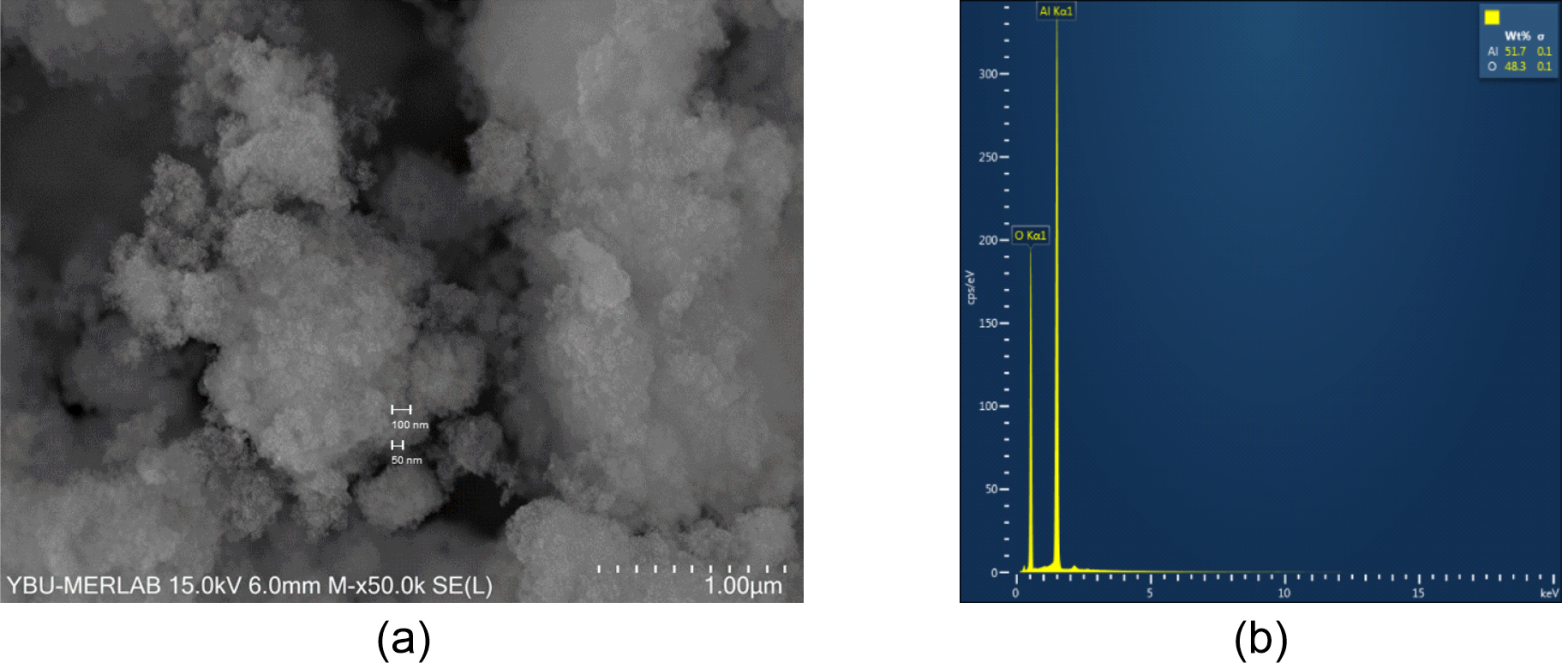
The a) FE-SEM micrograph and b) EDS analysis of Al_2_O_3_ nanoparticles.

The morphological features of the Al_2_O_3_ nanoparticles were investigated with the help of FE-SEM micrograph (Figure 2a). It is seen that the Al_2_O_3_ nanoparticles exhibit amorphous nature. Thus, it can be stated that the particle size cannot be clearly determined in FE-SEM micrograph, but the primary particle size is smaller than 50 nm. On the other hand, EDS analysis was conducted on the Al_2_O_3_ sample to determine the elemental composition and purity of Al_2_O_3_ nanoparticles. The result of the EDS analysis is given in Figure 2b and it reveals the presence of aluminium (Al) and oxygen (O) elements with weight percentages of 51.7 and 48.3, respectively. Using these weight percentages, the atomic percentages of Al and O are calculated to be 38.8 and 62.2, respectively. This indicates the presence of Al and O elements in the sample free from impurities. This provides strong evidence of the purity of the Al_2_O_3_ nanoparticles employed in the study. Also, it was seen that the EDS analysis result agrees with previous studies [22].

**Figure 3:**
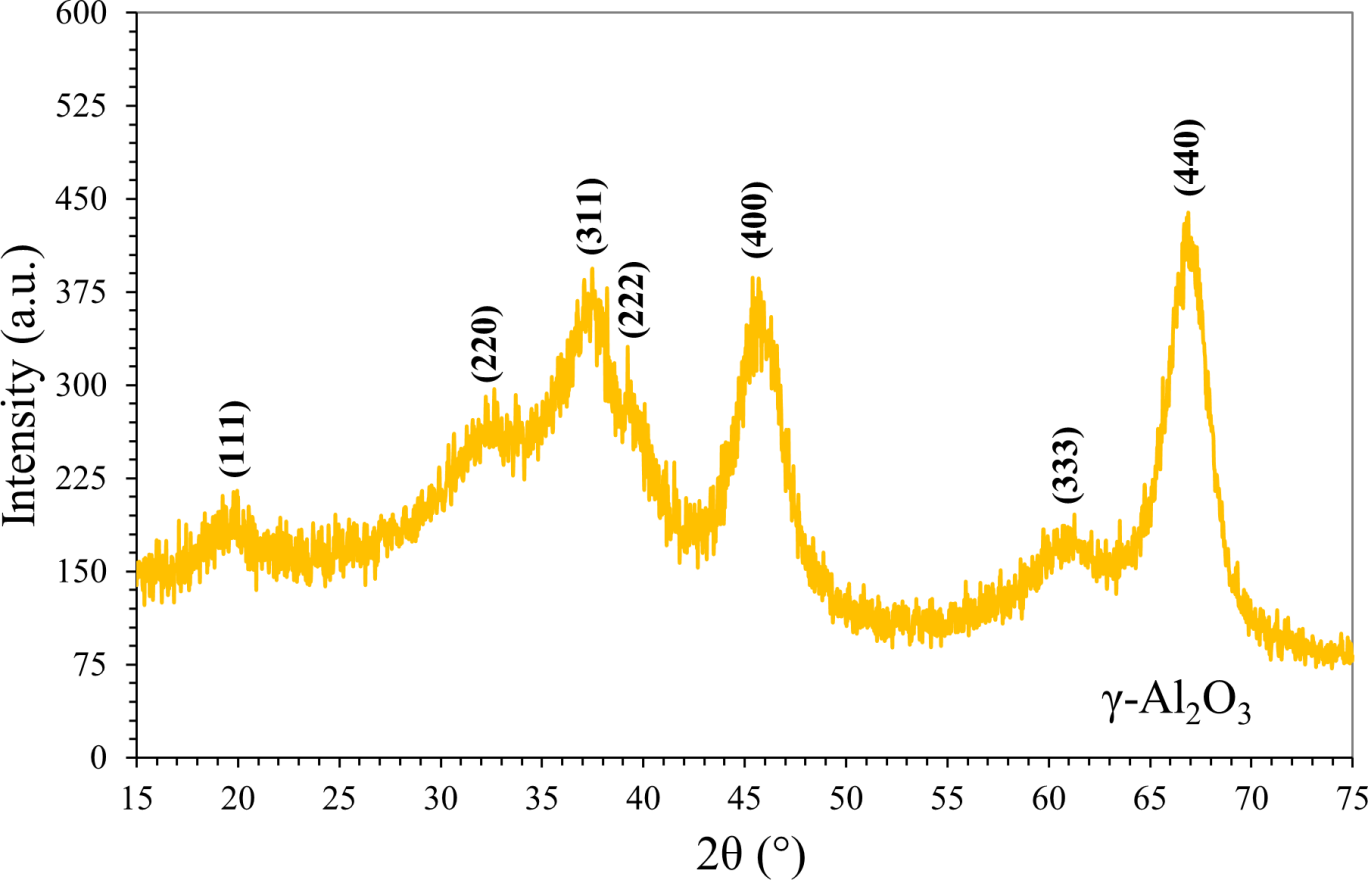
X-ray diffraction pattern of Al_2_O_3_ nanoparticles [21].

The crystalline properties of Al_2_O_3_ nanoparticle were determined by XRD analysis. All peaks were measured by XRD and compared with previous studies [23–24] in the literature. The XRD patterns of the Al_2_O_3_ nanoparticle samples were recorded in the range 2θ (15°–75°) at room temperature. The XRD pattern of γ-Al_2_O_3_ is shown in Figure 3. The diffraction pattern of γ-Al_2_O_3_ shows strong peaks at angle (2θ) of 19.5°, 32.2°, 37.4°, 39.7°, 45.8°, 60.5°, and 67.0° which are indexed to Miller indices (111), (220), (311), (222), (400), (333) and (440), respectively. These peak placements correspond nicely with previous studies. Furthermore, the broad peaks observed in the XRD patterns provide additional evidence of the amorphous nature of the material. It is noted that the Al_2_O_3_ nanoparticles used in this study have been previously evaluated and similar XRD results were reported in our previous study [21].

### Characterization of graphene nanoplatelets

**Figure 4:**
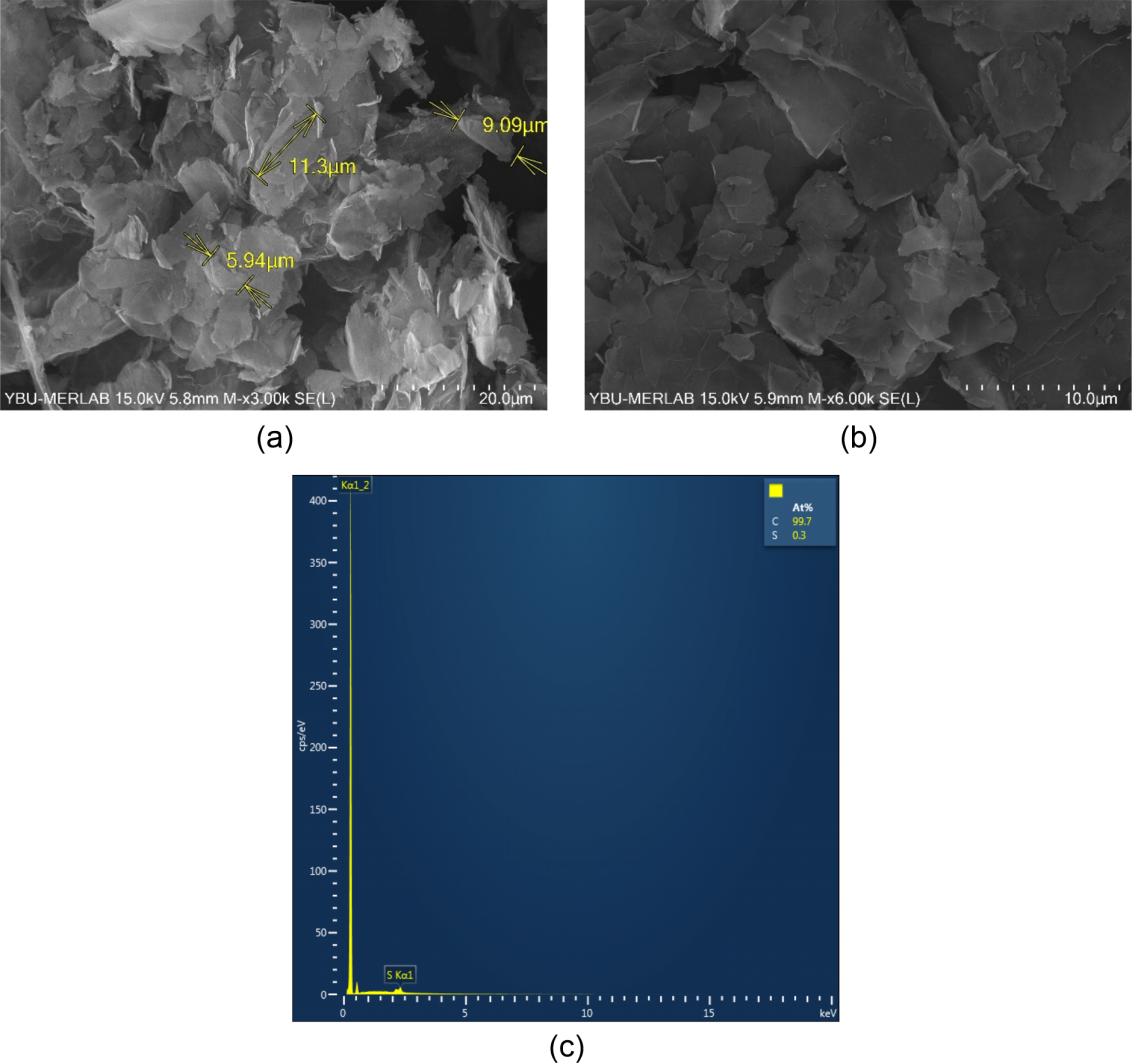
The a) FE-SEM micrograph with a scale bar of 20 µm, b) FE-SEM micrograph with a scale bar of 10 µm, and c) EDS analysis of the graphene nanoplatelets.

The morphological features of the graphene nanoparticles were evaluated using FE-SEM, and the micrographs obtained are given in Figure 4. Accordingly, Figure 4a and Figure 4b show the FE-SEM micrographs of the graphene used in this study with a scale bars of 20 and 10 µm, respectively. According to Figure 4a, the approximate diameter of 18 µm provided by the manufacturer for graphene nanomaterials (refer to Table 3) is consistent. The diameter value is a common characteristic value encountered in the literature for graphene nanomaterials [25]. Moreover, Figure 4b shows the multilayer structure of graphene nanoplates.

The EDS was performed on the graphene sample to determine the elemental composition and purity of the graphene nanomaterial. The results of the EDS analysis are given in Figure 4c, which reveals the existence of C and Selements, with atomic weight percentages of 99.7 and 0.3, respectively. The origin of the detected S, as revealed by EDS analysis, may be sulfuric acid which is utilized for activating the graphene in the graphene synthesis.

**Figure 5:**
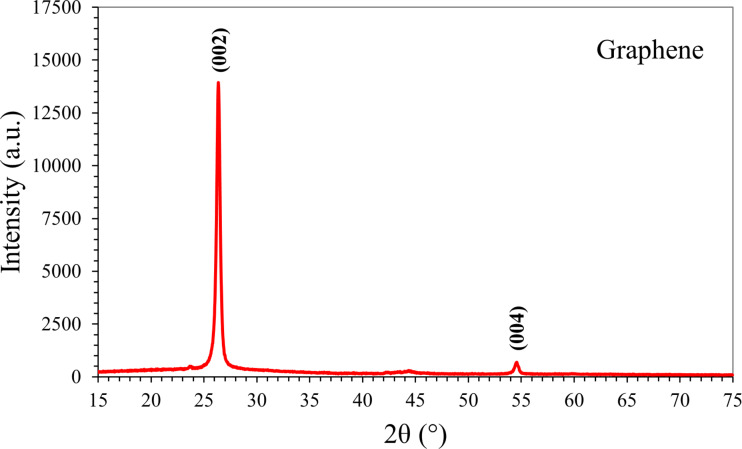
The XRD pattern of the graphene nanoplatelets.

The crystalline properties of graphene nanoplatelets were determined by XRD analyses. All peaks were measured by XRD and compared with previous studies [26]. The XRD patterns of the graphene nanoplatelet samples are recorded in the range of 2θ (15°–75°). The Figure 5 shows that there are two characteristic peaks in the XRD pattern of the graphene nanoplatelets (around 2θ = 27° and 2θ = 54°), which were assigned to the (002) and (004) plane. These peak placements nicely correspond with previous studies [26].

### Characterization of CNT nanoparticles

**Figure 6:**
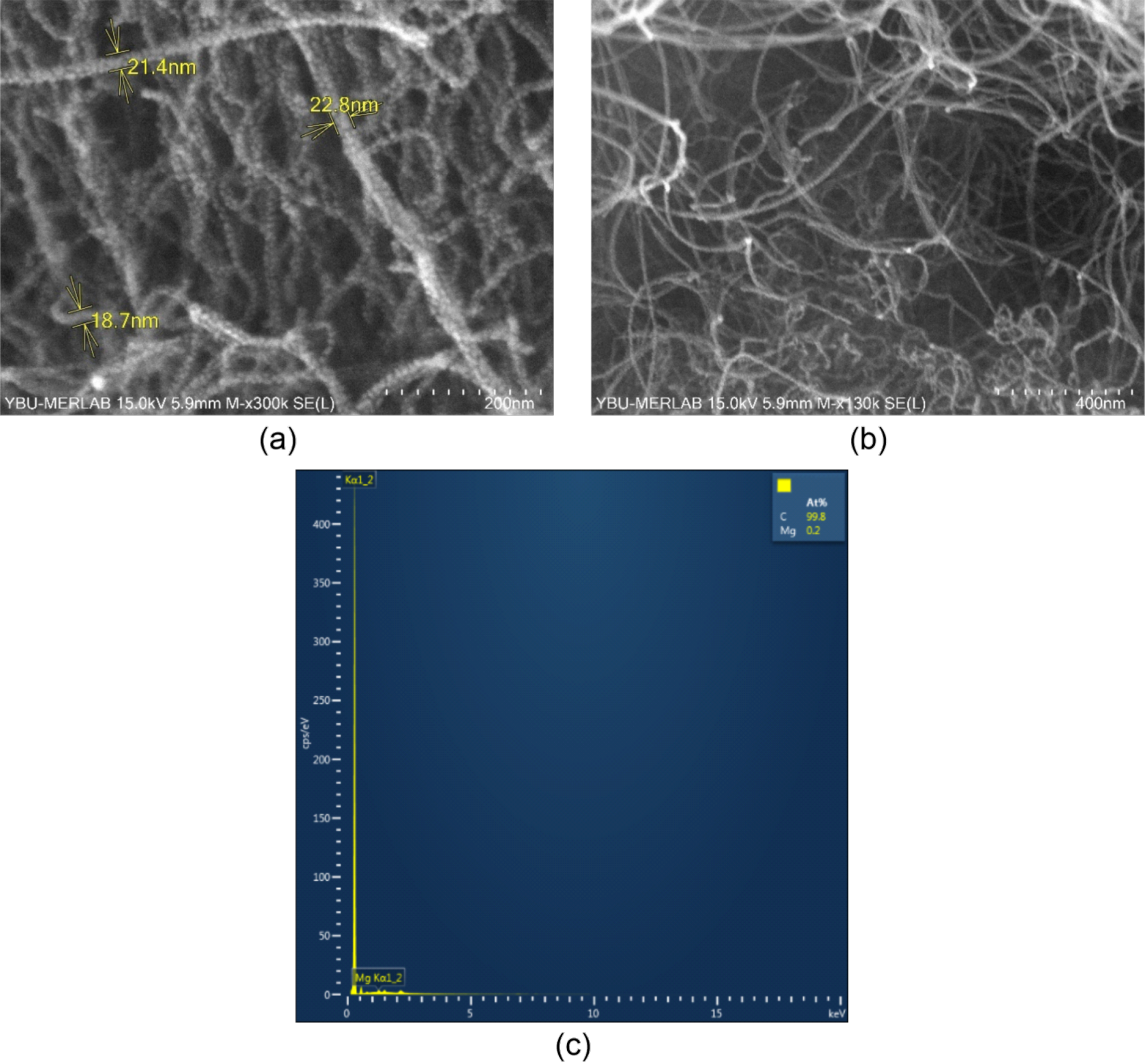
The a) FE-SEM micrograph with a scale bar of 200 nm, b) FE-SEM micrograph with a scale bar of 400 nm, and c) EDS analysis of the CNT nanoparticles.

The morphological properties of the CNT nanoparticles were evaluated using FE-SEM, and the micrographs obtained are given in Figure 6. Figure 6a and Figure 6b show the FE-SEM micrographs of the CNTs used in this study with scale bars of 200 and 400 nm, respectively. Figure 6a shows that the average diameter provided by the manufacturer presented in Table 3 is consistent. The manufacturer provides a length/diameter ratio of approximately 1000 (refer to Table 3) which is a commonly reported value in a previous study [27]. However, the length/diameter ratio of the sample cannot be accurately determined with the help of the FE-SEM micrographs due to the curved structure of the CNT. Nevertheless, the overlapping average diameter information corroborates the data provided by the manufacturer. Additionally, Figure 6b shows that the CNTs have a smooth surface with mixed tube bundles.

The EDS was conducted on the CNT sample to determine the elemental composition and purity of the CNT nanoparticles. The result of the EDS analysis is given in Figure 6c. Accordingly, the EDS analysis revealed the existence of C and Mg elements, with atomic weight percentages of 99.8 and 0.2, respectively. It is considered that the reason for the presence of 0.2% Mg in the EDS analysis may be due to the use of MgO as catalyst support [28].

**Figure 7:**
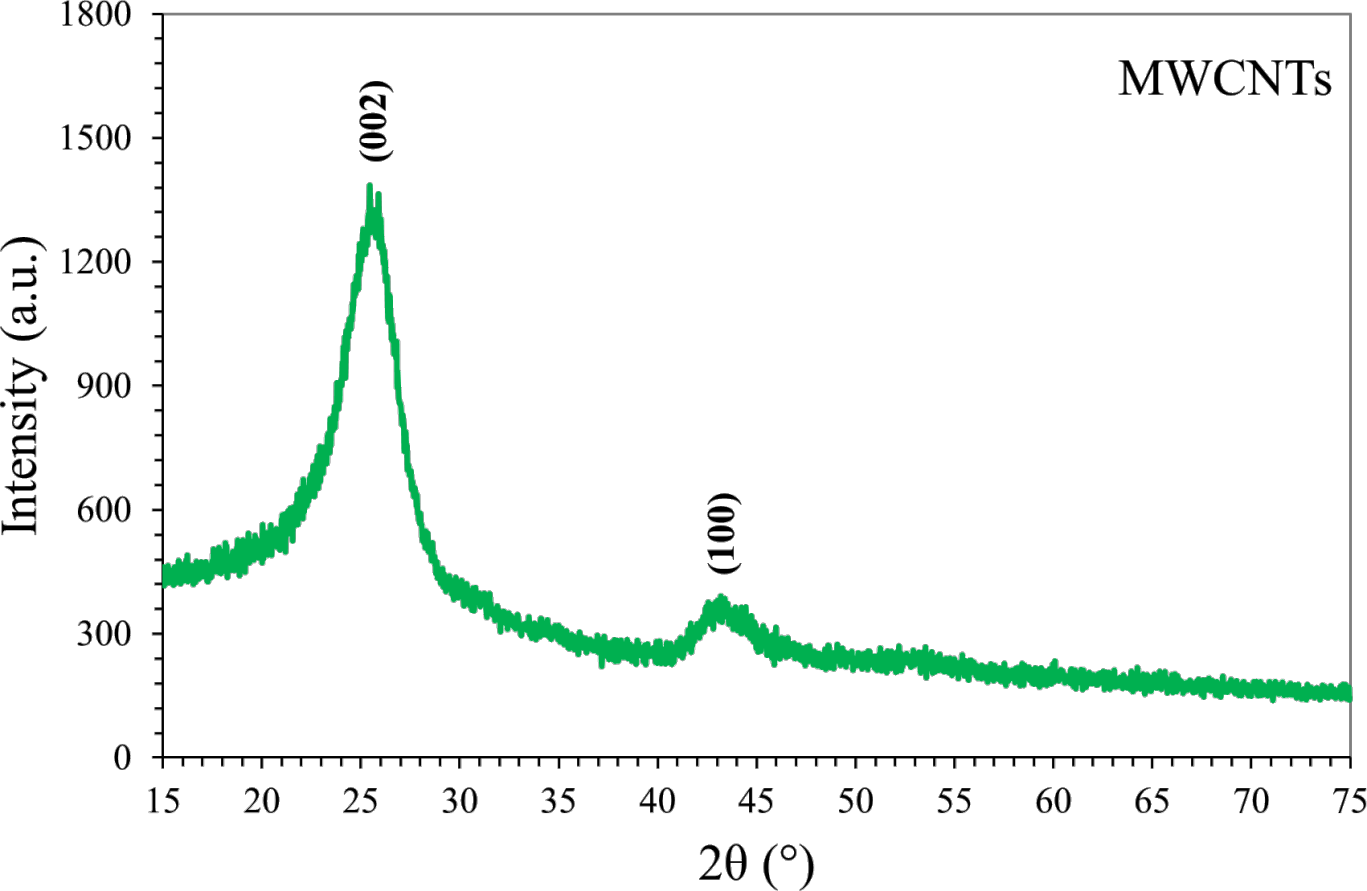
XRD pattern of the CNT nanoparticles.

The crystalline properties of CNTs nanoparticle were determined by XRD analysis. All peaks were measured by XRD and compared with previous studies [29–30] in the literature. The XRD patterns of the CNTs nanoparticle samples are recorded in the range 2θ (15°–75°). The Figure 7 depicts the XRD pattern of the purified CNTs in which diffraction peaks at 26° and 43° are corresponding to (002) and (100) reflection planes, respectively, which are indexed to the hexagonal graphite peak for the carbon nanotubes (JCPDS No. 41-1487). These peak placements correspond nicely with previous studies in the literature.

### Lubricant

In this study, nanoparticles were added to the compressor lubricant. The lubricant of the compressor in the test installation was an EMKARATE RL 32H POE oil compatible with the R1234yf refrigerant. The POE oil viscosity index, which is a measure of its working consistency over a wide temperature range is 121. This value indicates that the lubricant can consistently perform its lubricating function over a wide temperature range. Since the compressor operates in a wide temperature range in the operating conditions of the test installation (i.e., −10 to 80 °C), the importance of the viscosity index is even better understood. Another important property is the pour point temperature. The fluidity of the lubricant decreases with decreasing temperature and completely disappears at temperatures lower than the pour point temperature. The pour point temperature of the lubricant used in this study is −55 °C. For the tests in the experimental setup, the lowest temperature at which the system can go down to was about −10 °C. Therefore, the lowest temperature that the system will reach under operating conditions remains well above the lowest pour point temperature of the lubricant. The flash point temperature, which is another critical parameter for the safe operation of the system, was 264 °C. It is known that the maximum temperature that can be reached in the test installation is approximately 80 °C under standard operating conditions. In this respect, it is considered that the POE lubricant preferred within the scope of the study is an ideal lubricant for the R1234yf refrigerant used in the experimental setup. Technical information provided by the manufacturer about the POE oil is given in Table 4.

**Table 4:** Technical information provided by the manufacturer about the POE oil [31].

Properties	Method/standard	Typical value

viscosity	ASTM D445	32.5 cSt (at 40 °C)
viscosity	ASTM D445	5.8 cSt (at 100 °C)
viscosity index	ASTM D2270	121
pour point	ASTM D97	−55 °C
density	ASTM D1298	0.98 g/mL (at 20 °C)
flash point	ASTM D92	264 °C

### Two-step method

**Figure 8:**
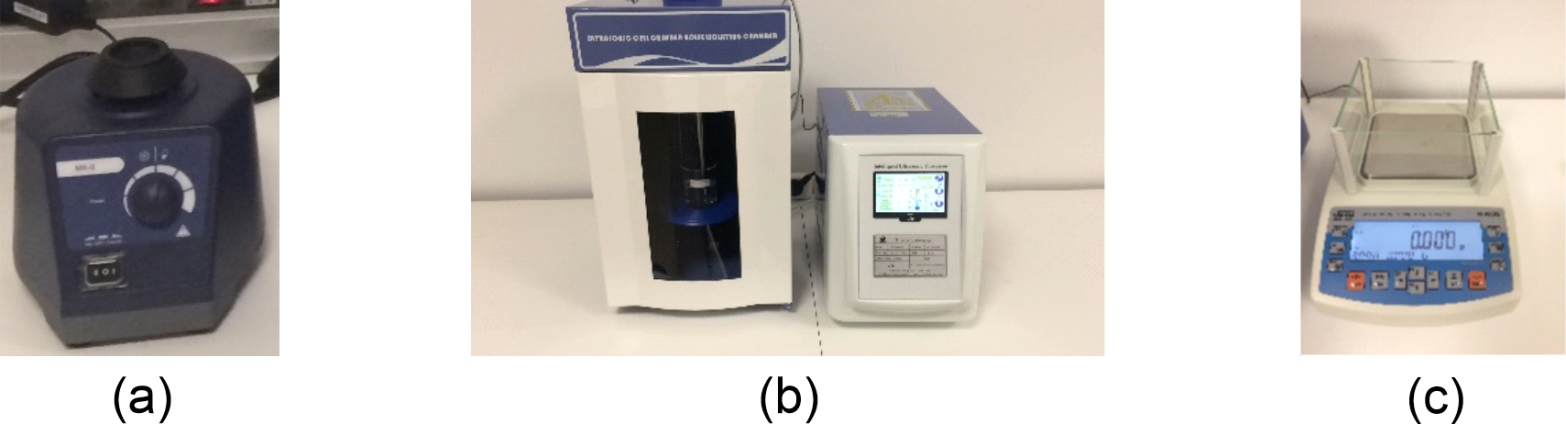
The devices used to implement the two-step method are a) mechanical stirrer, b) ultrasonic stirrer, and c) precision balance.

The two-step method, which is frequently preferred in the literature [12], was used for adding nanoparticles to the POE lubricant. The mass measurements of the samples were measured with a RADWAG PS1000.R2 precision balance. Firstly, the POE oil and nanoparticles were stirred in a mechanical stirrer, then the mixture was stirred with an ultrasonic stirrer. The mechanical mixing process was applied with the TOPTION MX-S mechanical mixer. Also, an ultrasonic mixing process was applied with the TOPTION TU-900E4 sonic mixer. The samples, which were kept in the sonic mixer for 90 minutes [32], were filled into the lubricant chamber of the compressor for tests. The devices used in the application of two-step method are shown in Figure 8.

### Stability of nanolubricants

**Figure 9:**
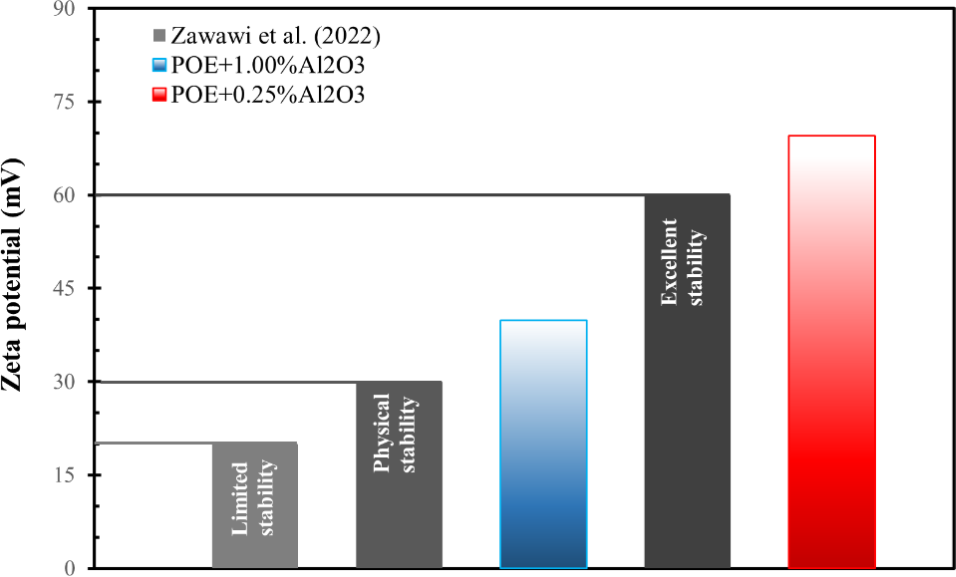
Zeta potential of nanolubricants containing Al_2_O_3_ at the lowest and highest mass fractions [21].

In the preparation process, the sonication time was 90 minutes to prevent sedimentation as suggested in previous studies [32]. The zeta potential values of nanolubricants containing Al_2_O_3_ were also measured and given in Figure 9. Accordingly, it can be said that the stability of nanolubricant samples with Al_2_O_3_ is excellent and normal for the lowest mass fraction (ω = 0.25%) and highest mass fraction (ω = 1.00%), respectively [33–34]. It is noted that data about the stability of the nanolubricant shown in Figure 9 has also been used in a previous study [21]. Nanolubricants containing graphene and CNTs are also considered to be stable since they are obtained by the same method.

### Determination of electrical power and kinematic viscosity

**Figure 10:**
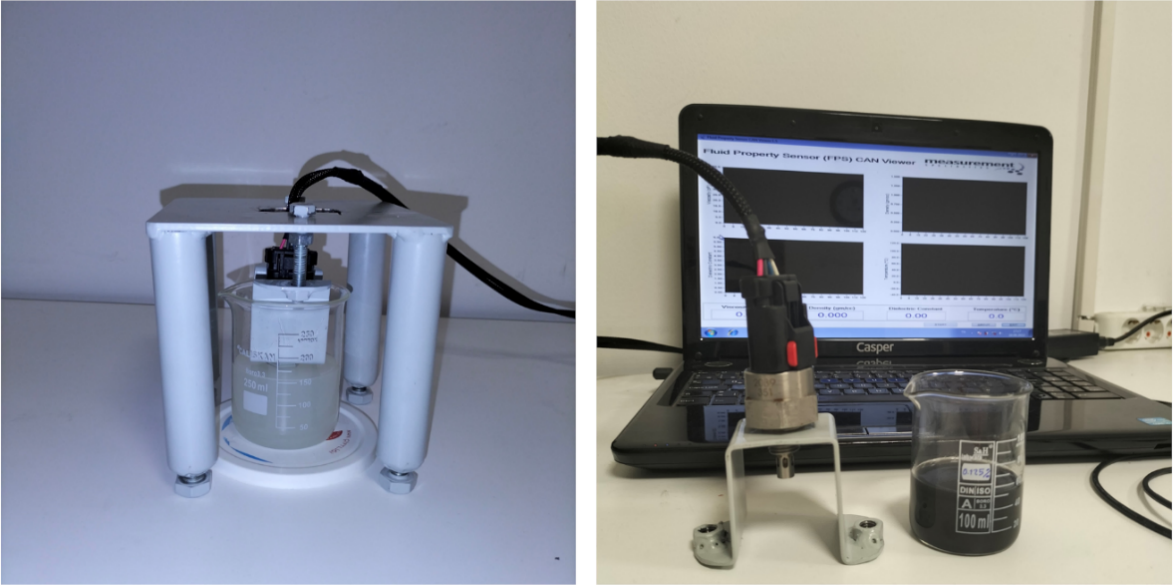
Images of the FPS2800 fluid property sensor.

Experiments in the refrigeration compressor were performed under steady-state test conditions. The required electrical power of the compressor, 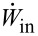, was directly measured by the digital wattmeter during the experiments. The kinematic viscosity, ν, was defined as the ratio of the dynamic viscosity to the density according to Equation 1. The density, ρ, of the nanolubricants was measured with the FPS2800 fluid property sensor. At the same time, the dynamic viscosity, µ, of the nanolubricants was measured using the FPS 2800 fluid property sensor. The technical information of the FPS2800 fluid property sensor is also given in Table 5. The images of the FPS2800 fluid property sensor are shown in Figure 10.


[1] $$\nu =\frac{\mu }{\rho }.$$

**Table 5:** Technical data of the FPS2800 fluid property sensor.

Property	Measuring range	Accuracy

density	0 to 1.5 g/cm^3^	±3%
dynamic viscosity	0 to 50 mPa·s	±5% (µ > 10 mPa·s) ±0.2 mPa·s (µ < 10 mPa·s)
temperature	−40 to +150 °C	±2 °C

### Uncertainty analysis

In this experimental study, the uncertainty analysis method was applied in the error analysis. In this method, *R* and *n* are the magnitudes calculated by means of any test setup and the number of independent variables affecting *R*, respectively. In this case, it can be written as *R* = *R*(*x*_1_, *x*_2_, *x*_3_, … *x_n_*). The uncertainties of the independent variables that are effective in the experiments are 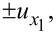 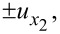 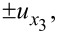 and 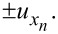 In this case, the uncertainty of *R* is expressed as Equation 2.


[2]

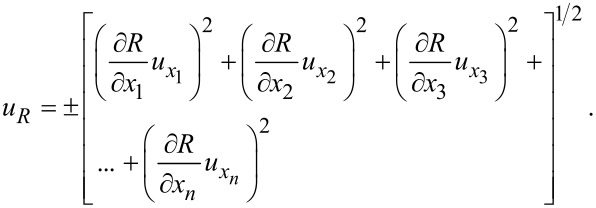



The only magnitude calculated based on the measurement data within the scope of this study is the kinematic viscosity value. Other magnitudes were determined by direct measurement, and the uncertainty of the measurement devices was considered in the error analysis. The kinematic viscosity is calculated by Equation 1 with the help of the data obtained by measuring the density and dynamic viscosity values. Therefore, there is a need to determine the effect of uncertainties in the measured data of the calculated kinematic viscosity value. Thus, the kinematic viscosity expression given in Equation 1 was adapted according to the uncertainty analysis relation given in Equation 2, and the uncertainty amounts for the kinematic viscosity calculations within the scope of this study were calculated with the help of Equation 3.


[3] $$u_{\nu }=\pm {\left[{\left(\frac{\partial \nu }{\partial \mu }u_{\mu }\right)}^{2}+{\left(\frac{\partial \nu }{\partial \rho }u_{\rho }\right)}^{2}\right]}^{1/2}.$$

The maximum uncertainty amount calculated with the help of Equation 3 for the kinematic viscosity value was ±5.83%. These calculated uncertainty amounts are indicated on the graphs in which the kinematic viscosity is expressed.

### Experimental procedure

The minimum mass fraction of Al_2_O_3_, graphene, and CNT nanoparticles in the POE oil was determined considering similar studies in the literature. Since Al_2_O_3_ is a metal oxide, mass fractions are different from graphene and CNTs, which are carbon allotropes. Thus, the minimum mass fractions were determined as 0.250% for Al_2_O_3_ [20] and 0.125% for both graphene and CNT nanoparticles [35]. Each nanoparticle type was gradually added to the pure POE oil starting from these minimum values. The compressor electrical power was controlled during the tests, and the nanoparticle mass fraction was increased as long as the performance enhancement was observed. Tests for the relevant nanoparticle type were stopped at the mass fraction where the compressor power input started to increase again. Then it was passed to another type of nanoparticles. Thus, it was aimed to determine the optimum mass fraction of nanoparticles.

## Results and Discussion

This study investigated the effects of using nanolubricants containing Al_2_O_3_, graphene, and CNTs on the required electrical power of a refrigeration compressor. Also, the kinematic viscosity of the nanolubricants was determined to gain a better physical understanding of the nanoparticle–lubricant mixture.

### Validation of test results

**Figure 11:**
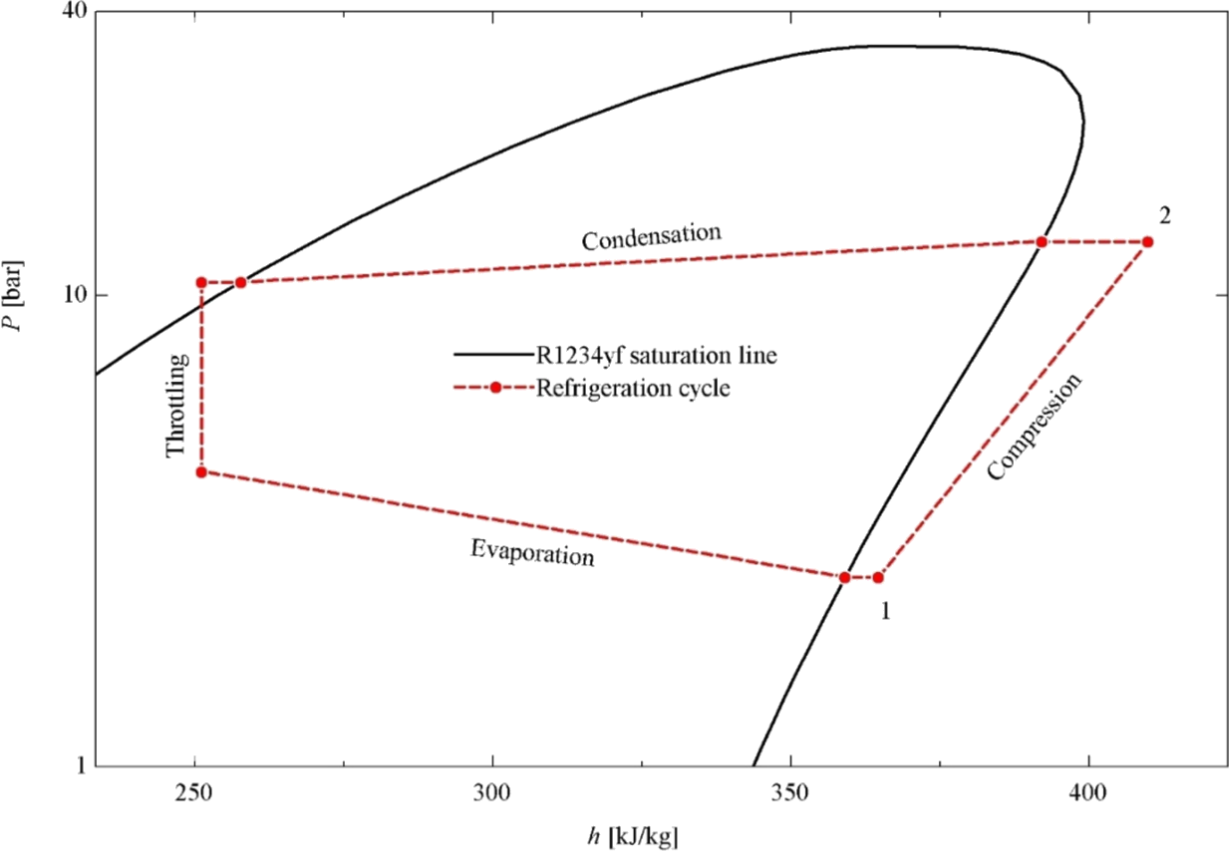
*P*–*h* diagram of the refrigeration system for validation tests.

The refrigeration compressor used in this study was selected in accordance with the R1234yf as the refrigerant. Superheating, which was applied to ensure that the refrigerant enters the compressor in gas phase, was approximately 6 °C. The superheating was controlled by an electronic expansion valve. The refrigerant was gradually charged to the system until the desired superheating value, and the tests were carried out when the superheating value reached the desired value. Thus, it was ensured that the amount of superheating was kept at the desired level in all tests. The tests were performed at approximately the same evaporation and condensation temperatures. To validate the study, the actual refrigeration cycle with R1234yf is presented as in previous studies [36]. The pressure (*P*) – specific enthalpy (*h*) diagram obtained from the validation tests was given in Figure 11. Accordingly, it was seen that the actual *P*–*h* diagram overlapped with previous studies [36]. Thus, it was concluded that the experimental setup was reliable.

**Figure 12:**
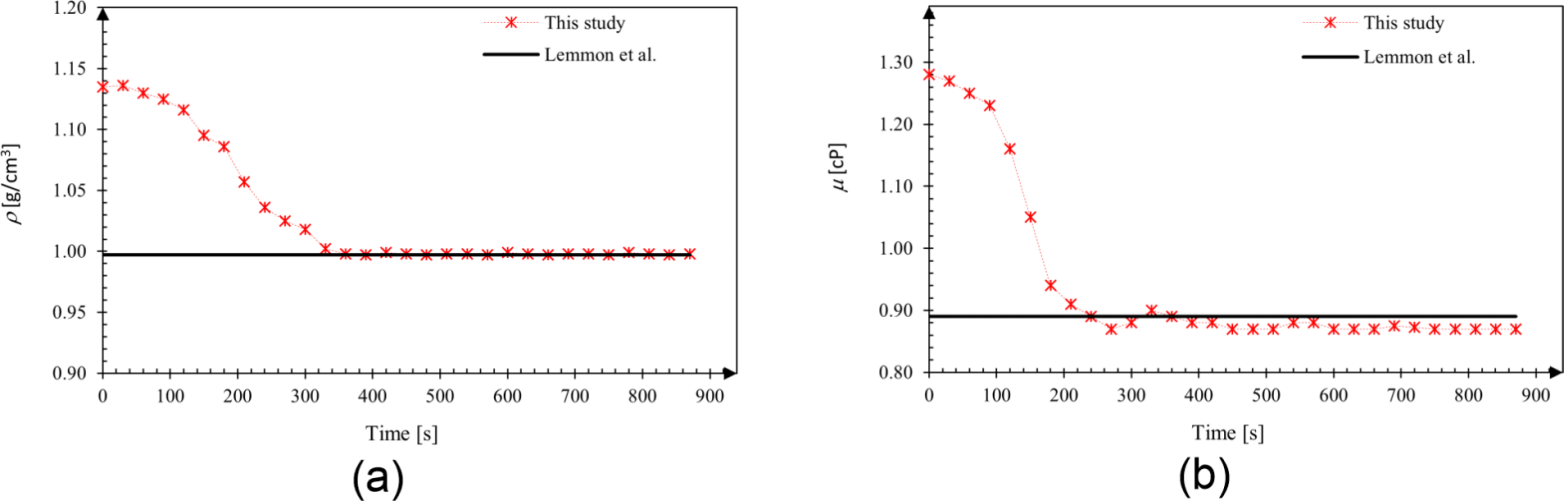
Measurement of a) density and b) dynamic viscosity of water for validation at 25 °C.

The verification of measurements of the FPS2800 fluid property sensor used in the determination of kinematic viscosity was also carried out with water. The density and dynamic viscosity of the water sample were measured at 25 °C and shown in Figure 12. The measurement results were compatible with the National Institute of Standards and Technology (NIST) data [37] at 25 °C.

### Results of compressor electrical power measurements

Compressors are devices used to compress any fluid that has a gas phase, and that requires a power input to perform this compression process. The same applies to refrigeration compressors. Therefore, a reduction in compressor power consumption means an increase in compressor performance. In this study, three types of nanoparticles, called Al_2_O_3_, graphene, and CNTs, were experimentally investigated in terms of compressor electrical power requirements at various mass fractions in the compressor oil. The nanoparticles were added to the compressor lubricant in a two-step method. During the experiment, the operating conditions in the pure oil tests were kept constant. Only the nanoparticle type and mass fraction were changed and the changes in the required compressor electrical power were recorded. In the tests, the nanoparticle mass fraction was gradually increased for each nanoparticle type under the operating conditions of the pure oil. In this way, the optimum fraction for each nanoparticle type and the best results among all cases were determined.

**Figure 13:**
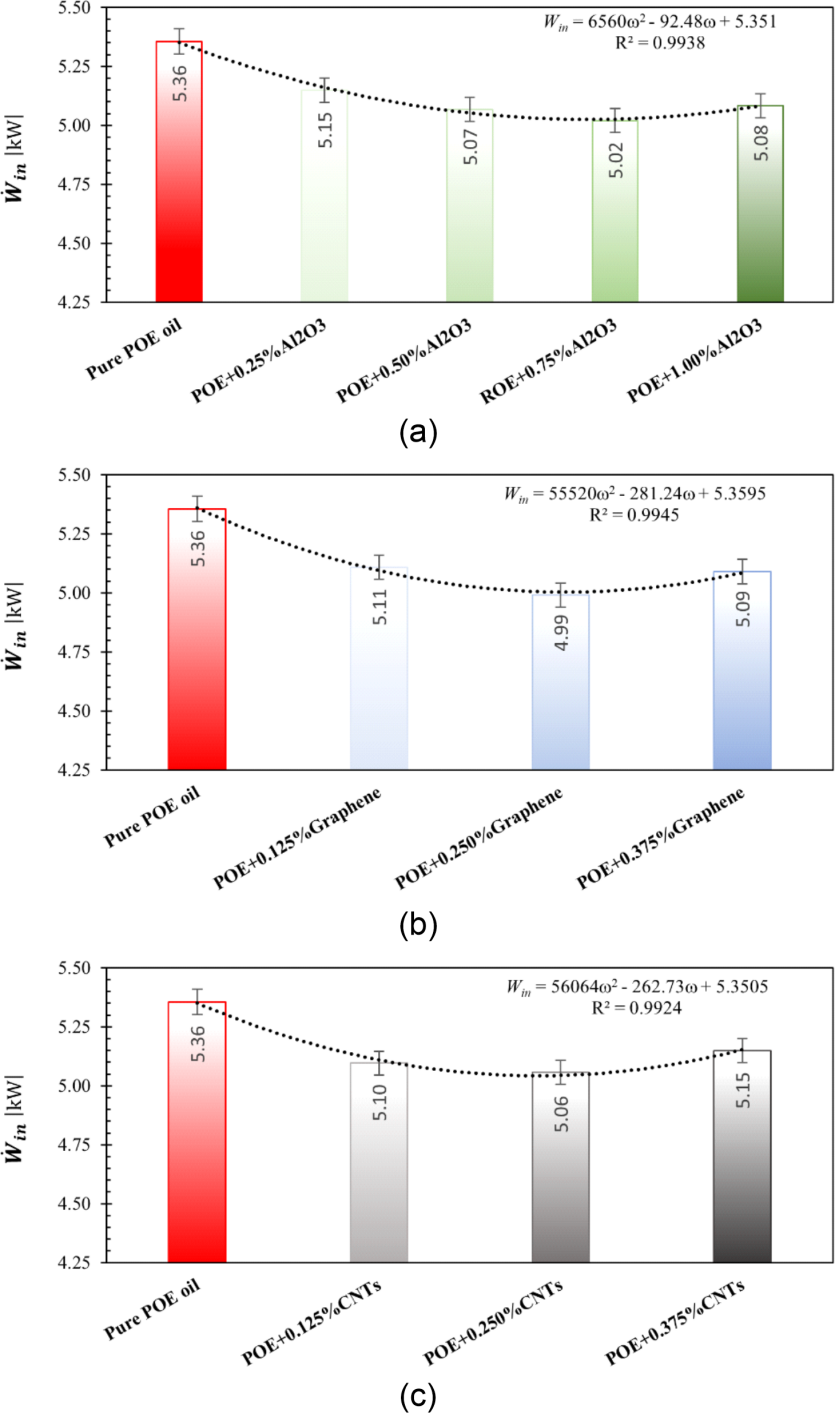
Required compressor electrical power for the usage of nanolubricants with a) Al_2_O_3_, b) graphene, and c) CNTs.

First of all, it should be noted that the refrigeration compressor used in this study safely operated with all nanolubricants. The required compressor electrical power of the compressor is shown in Figure 13a, Figure 13b, and Figure 13c for the use of nanolubricants containing Al_2_O_3_, graphene, and CNTs, respectively. Al_2_O_3_ nanoparticles were gradually added to the lubricant starting from a mass fraction of 0.25%. According to Figure 13a, as the Al_2_O_3_ mass fraction increases up to 0.75%, a decrease in the compressor electrical power required to maintain similar operating conditions was observed. It then started to increase again. The maximum decrease in compressor electrical power was obtained as 6.26% at a mass fraction of 0.75%. Therefore, it can be concluded that the optimum mass fraction for Al_2_O_3_ nanoparticles is 0.75%.

Since graphene (carbon-based) nanoparticles have different properties than those of Al_2_O_3_ (metal oxide-based) nanoparticles, they were added to the VCRS at different mass fractions. Therefore, graphene nanoparticles were gradually added to the lubricant starting from a mass fraction of 0.125%. As shown in Figure 13b, it was found that the compressor electrical power required to maintain the same operating conditions decreased as a function of the graphene mass fraction up to 0.250%, and then started to increase again. The maximum decrease in compressor electrical power was found to be 6.82% at a 0.250% mass fraction. Therefore, it can be stated that the optimum mass fraction for graphene nanoparticles is 0.250%.

As CNTs are also carbon-based nanoparticles such as graphene, they were gradually added to the lubricant at the same mass fractions as graphene, starting from a mass fraction of 0.125%. According to Figure 13c, it was observed that the compressor electrical power required to provide similar operating conditions decreased as the CNTs mass fraction reached up to 0.250%, and then increased again. The maximum reduction in compressor electrical power obtained with the addition of CNT nanoparticles compared to the use of pure oil was calculated to be 5.55% at 0.250% mass fraction. Hence, it can be stated that the optimum mass fraction for CNT nanoparticles is 0.250%. At the same time, compressor electrical power correlations based on experimental data of usage in nanolubricants in refrigeration compressors were also proposed for each nanoparticle type. It is hoped that these correlations will be useful in studies under similar operating conditions.

**Figure 14:**
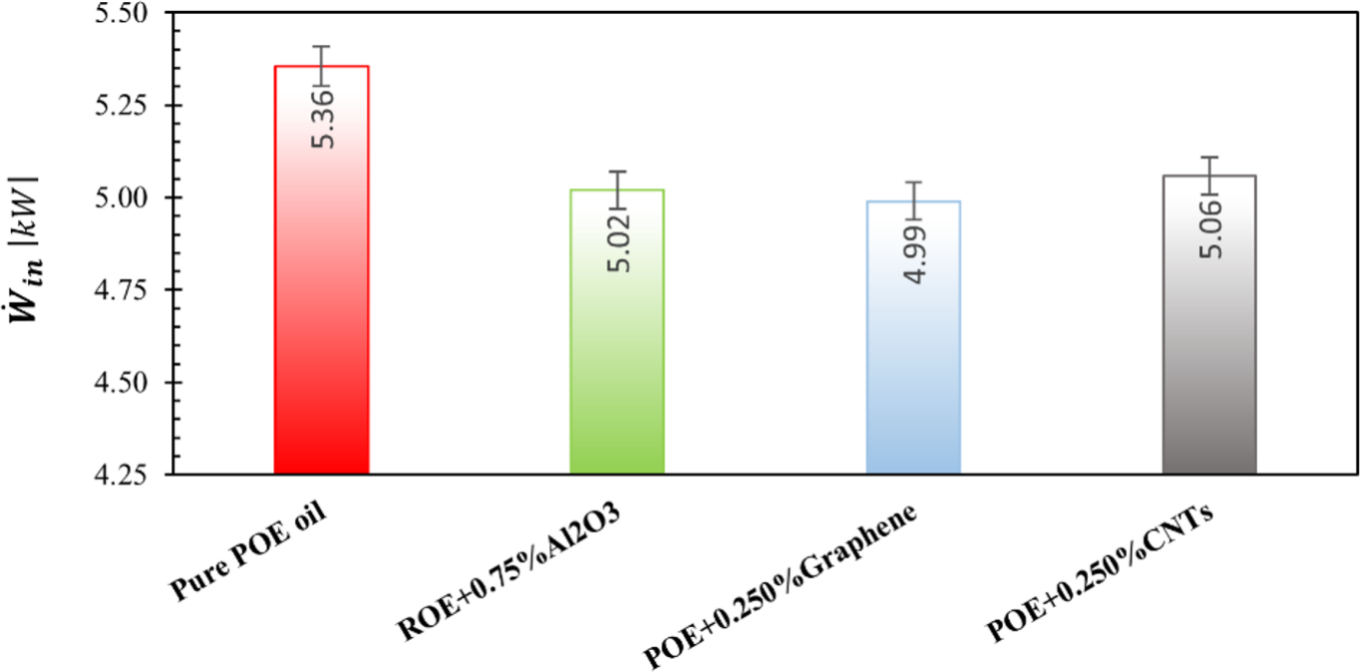
Required compressor electrical power for nanolubricants with optimum mass fraction of nanoparticles.

The use of nanolubricants with optimum nanoparticle mass fractions provides the best performance improvement by reducing the energy consumption of the compressor. The optimum mass fractions for the nanolubricants investigated in this study were 0.75, 0.25, and 0.25% for the nanolubricants containing Al_2_O_3_, graphene, and CNTs respectively. The compressor power consumption for nanolubricants with this determined optimum fraction is shown in Figure 14. Accordingly, the reductions in compressor power required due to the use of nanolubricants were determined to be approximately 6.26% for Al_2_O_3_, 6.82% for graphene, and 5.55% for CNT nanoparticles. It can be seen that the nanolubricant with the best performance increase is the POE+0.25% graphene.

**Figure 15:**
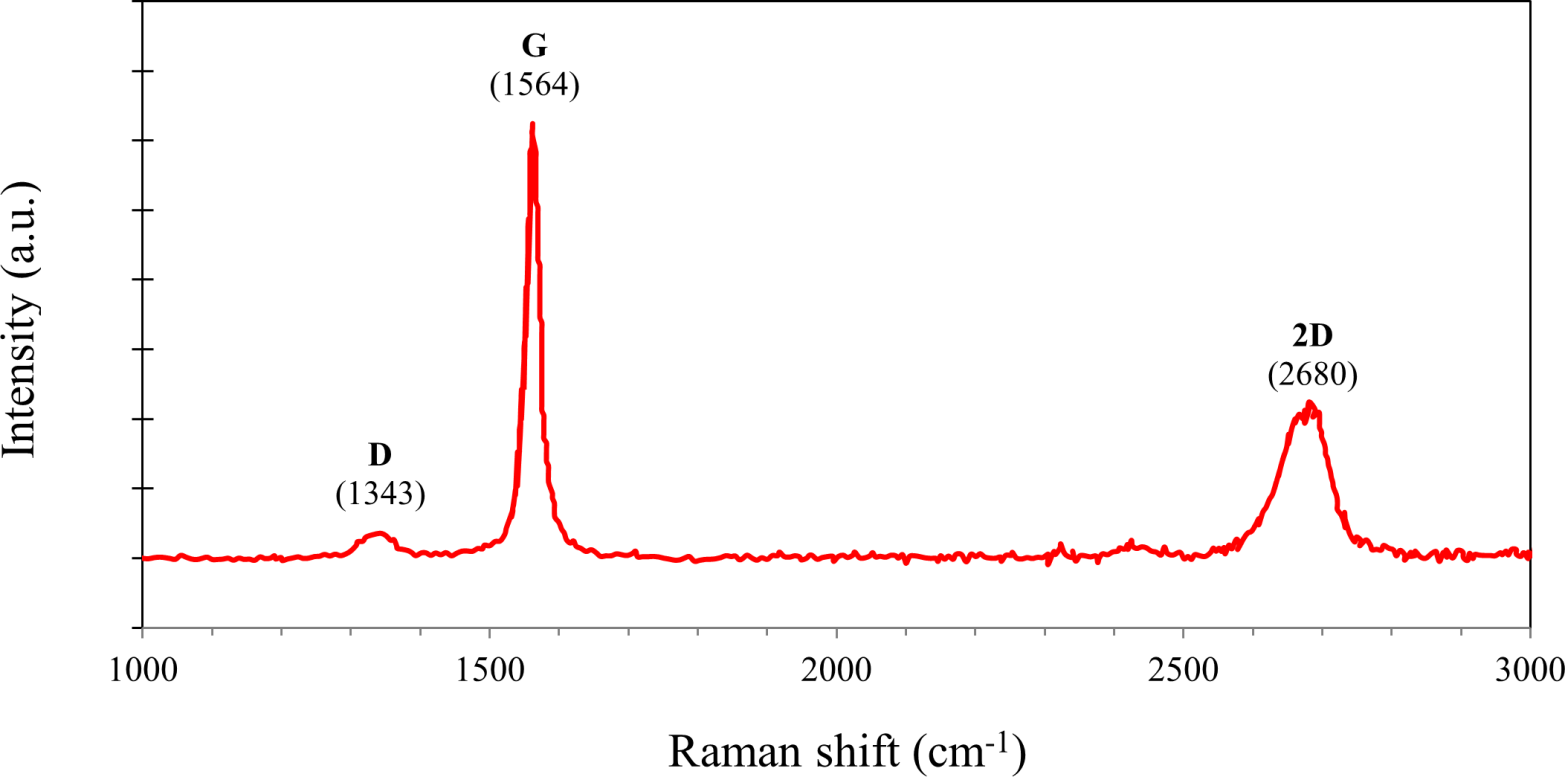
Raman spectroscopy of the graphene nanoplatelets.

The superior heat transport properties of graphene, when compared to those of Al_2_O_3_ and CNTs, is believed to be the reason for the effectiveness of a nanolubricant containing graphene nanoplatelets in the optimum mass fraction. The thermal conductivity coefficient of Al_2_O_3_ and CNTs are around 40 [38] and 3500 W/(m·K) [39], respectively. On the other hand, graphene has a thermal conductivity coefficient ranging from 4840 to 5300 W/(m·K) [39]. These data on thermal conductivity coefficients are applicable to pristine materials. However, impurities and defects of the materials strongly impact their heat transport behaviour. The characterisation of the materials discussed in the Methodology section did not reveal any obvious impurities or defects. Nevertheless, enriching the characterisation of the graphene nanoplatelets may facilitate the understanding of the dominant effect of graphene on compressor power. Thus, the Raman spectroscopy of graphene nanoplatelets which yield optimal enhancement in required compressor electrical power are presented in Figure 15. Figure 15 shows that the graphene used in this study has characteristic G (1564 cm^−1^) and 2D (2680 cm^−1^) bands. A low intensity D band (1343 cm^−1^) can also be seen. However, D/G < 1 indicates a multilayered graphene structure, which is also seen in the micrographs shown in Figure 4a and Figure 4b. The low intensity of the D band indicates low defect density [40].

**Figure 16:**
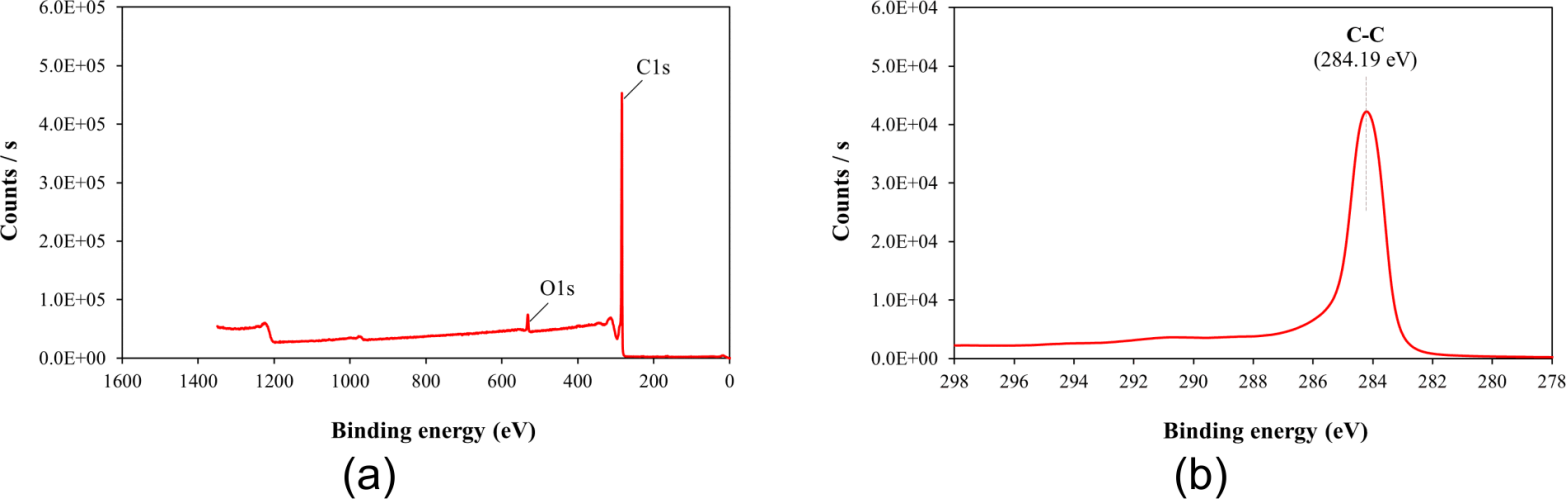
XPS of the graphene nanoplatelets.

The characteristic properties of graphene are directly related to the sp^2^ hybridisation in the chemical structure of the material. The chemical states of the elements for the graphene nanoplatelets were evaluated by XPS. Figure 16a shows a graphitic C 1s peak at 284.27 eV and a weak O 1s peak at 532.09 eV. Figure 16b shows that the graphene has a single intense peak at 284.19 eV corresponding to the sp^2^-hybridised C–C system. The XPS data obtained are also consistent with previous studies [41–42]. As a result, the high purity and low defect structures of graphene nanoplatelets used in this study are understood. This supports the fact that the best improvement occurs in the graphene-containing case.

### Results of viscosity measurement

The literature indicates that the use of nanolubricants reduces power consumption and the coefficient of friction compared to the use of pure lubricant [43]. However, the addition of nanoparticles to the lubricant above the optimum mass fraction had a negative effect on compressor performance. It is considered that the reason for this negative effect was the continuous increase in the density of the nanolubricant as the nanoparticle fraction increased. The fact that the addition of nanoparticles increased the density is shown in Table 6. According to the analytically obtained Equation 4, the density of the nanolubricant approaches the density of the nanoparticle as the fraction increases.

**Table 6:** Relation of nanolubricant density.

	POE oil	Nanoparticle	Nanolubricant

density	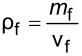	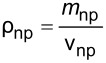	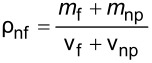

1st step	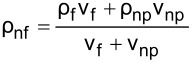		
2nd step	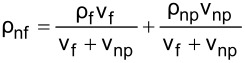		
3rd step	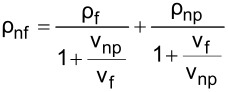		
4th step	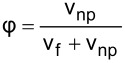		
5th step	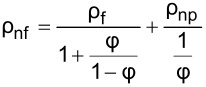		
6th step	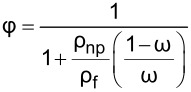		
final step	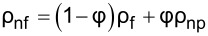		


[4] $$\rho_{\text{nf}}=\left(1-\varphi \right)\rho_{\text{f}}+\varphi \rho_{\text{np}}.$$

In Equation 4, ρ_nf_ is the nanolubricant density (g/cm^3^), ρ_f_ is the POE oil density (g/cm^3^), ρ_np_ is the nanoparticle density (g/cm^3^), φ represents the nanoparticle volume fraction (cm^3^-nanoparticles/cm^3^-nanolubricant), and ω the nanoparticle mass fraction (g-nanoparticles/g-nanolubricant).

**Figure 17:**
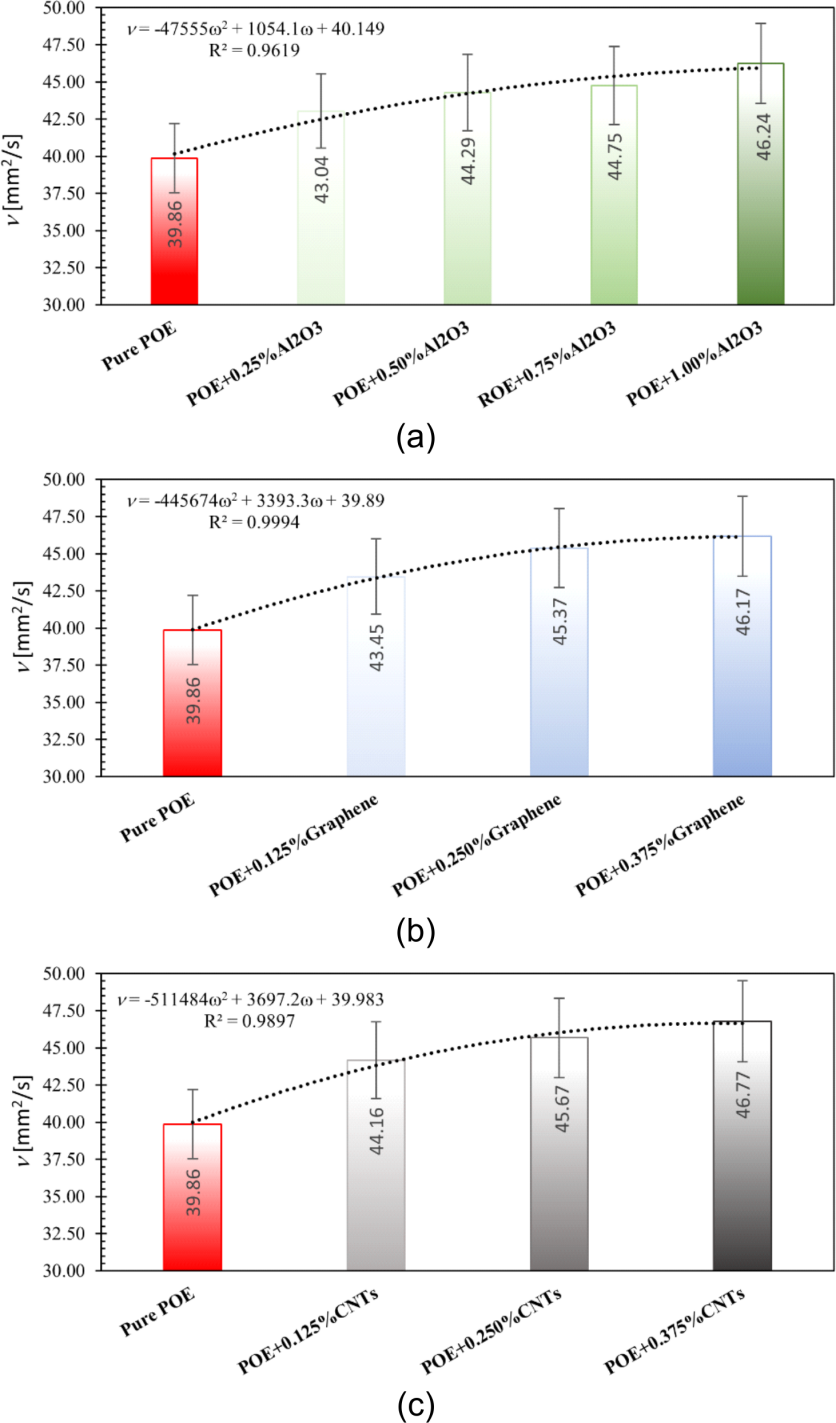
Kinematic viscosity of nanolubricants with a) Al_2_O_3_, b) graphene, and c) CNTs.

The addition of nanoparticles also affects the viscosity of the nanolubricant. The kinematic viscosity is given in Figure 17a, Figure 17b, and Figure 17c for the nanolubricants containing Al_2_O_3_, graphene, and CNTs, respectively. Accordingly, it was observed that the viscosity of the nanolubricant increased as the nanoparticle mass fraction increased. The increase in kinematic viscosity causes the viscous effects of the lubricant to become increasingly effective. Therefore, the viscous effects become dominant in the compressor, and the performance degradation begins when the optimum nanoparticle fraction is exceeded. On the other hand, kinematic viscosity is used to determine the viscosity index, which is one of the most important indicators of lubricant performance. For kinematic viscosity, correlations based on experimental results for each type of nanoparticle have been proposed for researchers who do not have the opportunity to conduct experimental studies. It is hoped that these correlations will be useful in studies under similar operating conditions.

**Figure 18:**
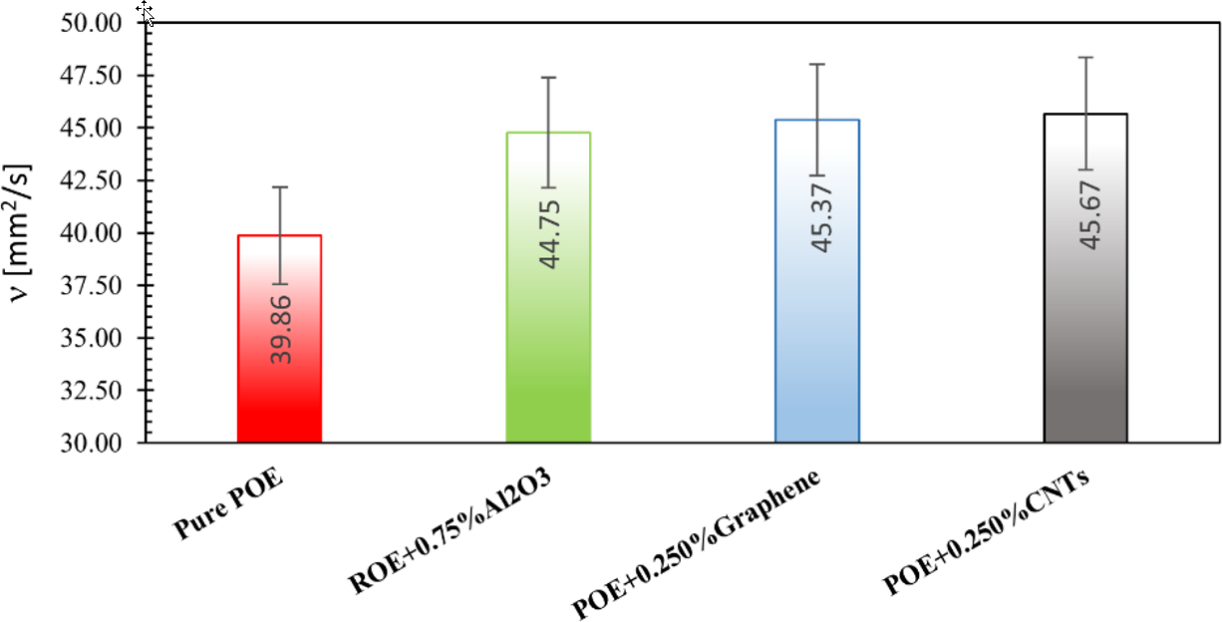
Kinematic viscosity of nanolubricants with optimum fraction.

The kinematic viscosity for nanolubricants with the determined optimum fraction is shown in Figure 18. The increase in viscosity due to the addition of nanoparticles was found to be approximately 12.27% for Al_2_O_3_, 13.82% for graphene, and 14.58% for CNT nanoparticles. Accordingly, it should be noted that carbon-based nanoparticles cause a greater increase in viscosity compared to Al_2_O_3_ nanoparticles, although they were added to the oil in a lower fraction.

## Conclusion

In this study, the effects of using nanolubricants containing Al_2_O_3_, graphene, and CNTs on the performance of a refrigeration compressor were experimentally investigated. In addition, the variation of the kinematic viscosity of nanolubricants with nanoparticle mass fraction was studied to physically understand the mixture of nanoparticles and lubricant. It was shown that he refrigeration compressor worked safely and efficiently with the nanolubricant. In addition, it was observed that the use of the nanolubricant improved the compressor performance up to a certain fraction of nanoparticles. The optimum mass fractions for the operating conditions of this study were 0.75% for Al_2_O_3_, 0.250% for graphene, and 0.250% for CNTs. The required compressor electrical power decreased by 6.26, 6.82, and 5.55% with the addition of Al_2_O_3_, graphene, and CNT nanoparticles at optimum mass fractions of 0.75, 0.250, and 0.250%, respectively, with the use of nanolubricants compared to the use of pure oil. The best improvement in the required electrical power of the compressor was obtained with the addition of graphene at an optimum mass fraction of 0.250%. Last but not least, the kinematic viscosity of the nanolubricant increased with increasing nanoparticle mass fraction.

It is clear that the use of nanolubricants has a positive effect on the performance of refrigeration compressors. However, it is very important that the nanoparticles are used in optimum fractions. Otherwise, the desired level of performance improvement will not be achieved and might result in additional costs. It is also noted that the performance of the refrigeration compressor depends on the nanoparticles used and lubricant type. For this reason, it is recommended to increase the number of studies including different types of lubricants and nanoparticles.
